# Virtual Digital Psychotherapist App–Based Treatment in Patients With Methamphetamine Use Disorder (Echo-APP): Single-Arm Pilot Feasibility and Efficacy Study

**DOI:** 10.2196/40373

**Published:** 2023-01-31

**Authors:** Tianzhen Chen, Liyu Chen, Shuo Li, Jiang Du, Hang Su, Haifeng Jiang, Qianying Wu, Lei Zhang, Jiayi Bao, Min Zhao

**Affiliations:** 1 Shanghai Mental Health Center Shanghai Jiao Tong University School of Medicine Shanghai China; 2 Shanghai Key Laboratory of Psychotic Disorders Shanghai China; 3 Center for Excellence in Brain Science and Intelligence Technology (CEBSIT) Chinese Academy of Sciences Shanghai China

**Keywords:** tablet, Android program, substance use disorder, methamphetamine use disorder, digital agent, virtual digital human

## Abstract

**Background:**

Substance use disorder is one of the severe public health problems worldwide. Inequitable resources, discrimination, and physical distances limit patients’ access to medical help. Automated conversational agents have the potential to provide in-home and remote therapy. However, automatic dialogue agents mostly use text and other methods to interact, which affects the interaction experience, treatment immersion, and clinical efficacy.

**Objective:**

The aim of this paper is to describe the design and development of Echo-APP, a tablet-based app with the function of a virtual digital psychotherapist, and to conduct a pilot study to explore the feasibility and preliminary efficacy results of Echo-APP for patients with methamphetamine use disorder.

**Methods:**

Echo-APP is an assessment and rehabilitation program developed for substance use disorder (SUD) by a team of clinicians, psychotherapists, and computer experts. The program is available for Android tablets. In terms of assessment, the focus is on the core characteristics of SUD, such as mood, impulsivity, treatment motivation, and craving level. In terms of treatment, Echo-APP provides 10 treatment units, involving awareness of addiction, motivation enhancement, emotion regulation, meditation, etc. A total of 47 patients with methamphetamine dependence were eventually enrolled in the pilot study to receive a single session of the Echo-APP–based motivational enhancement treatment. The outcomes were assessed before and after the patients’ treatment, including treatment motivation, craving levels, self-perception on the importance of drug abstinence, and their confidence in stopping the drug use.

**Results:**

In the pilot study, scores on the Stages of Change Readiness and Treatment Eagerness Scale and the questionnaire on motivation for abstaining from drugs significantly increased after the Echo-APP–based treatment (*P*<.001, Cohen *d*=–0.60), while craving was reduced (*P*=.01, Cohen *d*=0.38). Patients’ baseline Generalized Anxiety Disorder-7 assessment score (*β*=3.57; *P*<.001; 95% CI 0.80, 2.89) and Barratt Impulsiveness Scale (BIS)–motor impulsiveness score (*β*=–2.10; *P*=.04; 95% CI –0.94, –0.02) were predictive of changes in the patients’ treatment motivation during treatment. Moreover, patients’ baseline Generalized Anxiety Disorder-7 assessment score (*β*=–1.607; *P*=.03; 95% CI –3.08, –0.14), BIS—attentional impulsivity score (*β*=–2.43; *P*=.004; 95% CI –4.03, –0.83), and BIS—nonplanning impulsivity score (*β*=2.54; *P*=.002; 95% CI 0.98, 4.10) were predictive of changes in craving scores during treatment.

**Conclusions:**

Echo-APP is a practical, accepted, and promising virtual digital psychotherapist program for patients with methamphetamine dependence. The preliminary findings lay a good foundation for further optimization of the program and the promotion of large-scale randomized controlled clinical studies for SUD.

## Introduction

Substance abuse and dependence seriously endanger public health worldwide. Globally, 3.5 million people die from alcohol and illicit drug abuse each year [1]. China is also facing huge challenges brought on by illegal drugs, tobacco, and alcohol. According to the latest report, there are more than 300 million tobacco users, 123 million people drink alcohol excessively, and 1.8 million people use illegal drugs in China [2-4]. Substance dependence brings a series of physical and psychological damage, resulting in a large economic burden of disease. Psychotherapy is one of the most important treatment methods for drug dependence currently, including cognitive behavioral therapy (CBT), meditation, motivational enhancement therapy (MET) [5,6]. It can help improve treatment motivation, ameliorate emotional disorders, and decrease cravings that patients face. However, many people with substance dependence do not receive proper treatment, and less than 20% of patients have received standard treatment [7]. The main reasons include shortage of professionals, fear of discrimination, and economic and transportation constraints.

Artificial intelligence (AI) and robotics can help solve the substance dependence treatment dilemma. These technologies have been gradually applied to various mental health scenarios such as emotion regulation, evaluation and treatment of mental diseases, treatment efficacy prediction, and rehabilitation management. By simulating the “intelligent brain,” an intelligent machine or program that responds in a manner similar to human intelligence can simulate psychotherapists in the field of psychological assessment, diagnosis, and treatment, which can save labor costs, realize remote intervention, and improve professional use. In the past period, automatic conversational agents have received attention [8]. Automated conversational agents can provide services similar to a therapist or physician, but without the need for human assistance. Studies have shown that text-based dialogue agents have good user engagement and are effective in the treatment of mental symptoms [9-11]. By playing the role of “psychotherapists,“ AI devices reduce the patient's sense of shame and fear of being discriminated against and increase the possibility of patients revealing their true feelings [12]. On the other hand, the widespread use of the internet and intelligent terminals makes electronic medical care based on apps and AI technology have good application prospects. In 2021, the number of smartphone-owning users in China reached 950 million [13]. The development of a mental health program suitable for mobile smart terminals will further enhance the interest of users.

AI has been applied in related fields such as insomnia, anxiety, depression, schizophrenia, substance dependence, and other diseases [14-18]. Among them, some programs are self-administered, that is, after self-assessment, the program provides mental health–related education [19]. Some programs are conversational agents. Research evidence for conversational agent interventions for addressing psychological problems is growing rapidly and has the potential in terms of acceptability and effectiveness [12]. In substance dependence studies, digital interventions have also been found to reduce substance use behaviors [20] and have the potential to reduce the economic burden of disease from substance use disorder (SUD). These apps mainly use text, animation, or dialogue to provide services to patients [8]. Although these programs are reported to be effective, there are still challenges such as poor interaction experience and high dropout rate due to the lack of a more realistic image. Recent studies suggested a potential improvement in treatment effects when incorporated with the virtual image. For instance, Yokotani and colleagues [21] found that virtual agent (with digital image) has advantages in participants’ disclosure of sex-related symptoms. The study by Philip [22] suggested that the smartphone-based virtual agent is feasible in screening patients with sleep complaints and provides acceptable behavior advice [22]. Virtual agents that show better look and feel achieve better user experience [23]. However, most of the virtual agent psychotherapists (with digital image) currently constructed are not vivid enough, with rigid expressions and movements. Besides, few studies have used computer techniques to construct a virtual digital psychotherapist image to provide psychological support services in the field of substance use disorder.

Based on these considerations, we propose, design, and develop a mobile- or tablet-based app (Echo-APP) for the assessment and treatment of substance dependence. Echo-APP, which features a virtual digital human image of a psychotherapist, is a key-based interactive conversational agent program that provides general mental health education, addiction-related symptom tracking and recording, and customized comprehensive psychotherapy. In this study, we describe the development and design of Echo-APP and aim to evaluate the feasibility and preliminary efficacy of Echo-APP through a single-arm pre-post–treatment design.

## Methods

### Development of a Virtual Digital Psychotherapist App (Echo-APP)

The design and development of Echo-APP started in 2019. The app development was proposed by the Addiction Research Group of the Shanghai Mental Health Center and was optimized through discussions with clinicians, patients with drug dependence, technician, and computer scientists from Mofa Technology Corporation. The multidisciplinary team of this project team meets regularly to form collaborations based on the necessary development techniques and patient-centered design-centric tenet. The core development period is from June 2019 to August 2020, and the first version of the app is finalized. The Echo-APP currently developed is in Chinese.

**Figure 1:**
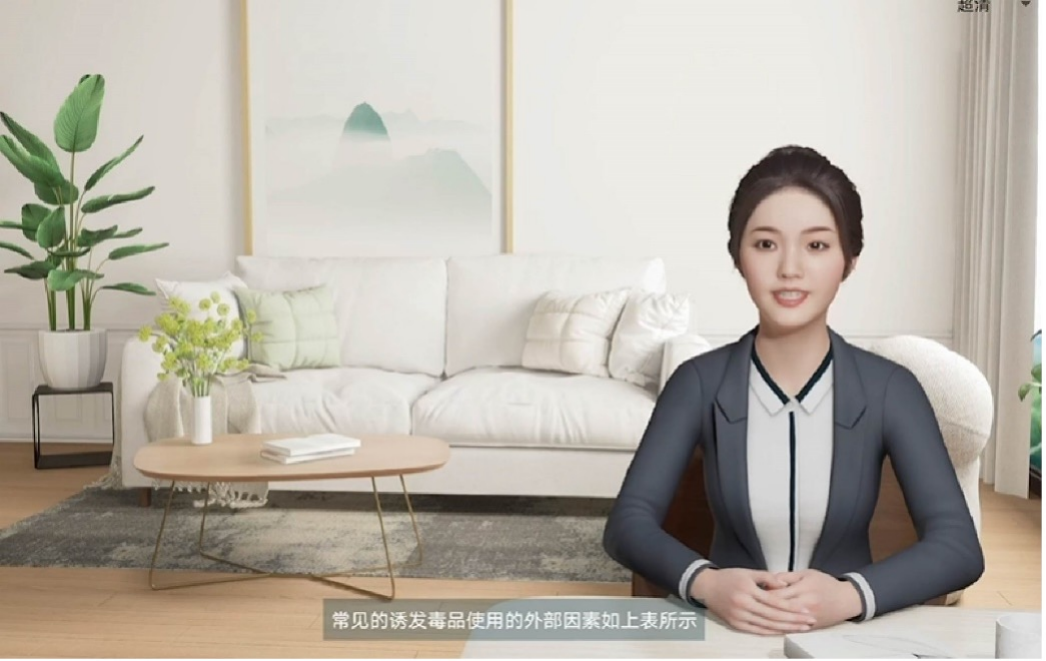
Image of the virtual digital psychotherapist in Echo-APP.

Echo-APP has been developed for the HUAWEI Android tablet above the application programming interface level 14 (version 4.0). Java (Oracle Corporation) and Python (Python Software Foundation) were used as the programming language, and Visual Studio (Microsoft Corporation) was used as the main development tool. In addition, the image of the virtual digital psychotherapist is developed based on digital human technology. Operationally, the main menu allows psychotherapists or social workers to add new subjects and refer to patients’ assessment and treatment records. A unique code is added to each patient. Three modules of “Psychological Assessment,” “Treatment Options,” and “Homework” are accessed via the buttons on the menu, ”Start Assessment,“ ”Start Therapy,“ and ”Homework.“ The process of assessment and treatment is completed by the virtual digital psychotherapist interacting with the patient. The image of the virtual digital psychotherapist (named “Xiaoying”) is shown in Figure 1.

### Assessment Module

The current version of Echo-APP could investigate patients' baseline demographic information, drug use history, emotional state, impulsivity trait, and treatment motivation. The interface of the assessment module is shown in Multimedia Appendix 1.

Demographic information is collected by self-designed scales, including the patient's age, gender, education level, employment status, and drug use characteristics.

The patient's emotional status is assessed by Generalized Anxiety Disorder-7 (GAD-7) and Patient Health Questionnaire-9 (PHQ-9).

Impulsivity characteristics are assessed by the Barratt Impulsiveness Scale (BIS)–11. BIS-11 has 30 items and can be divided into 3 dimensions (attention impulsivity, motor impulsivity, and nonplanning impulsivity).

Treatment motivation is assessed by the Stages of Change Readiness and Treatment Eagerness Scale (SOCRATES), which is self-assessed. This scale evaluates the treatment motivation from the following 3 aspects: patients’ awareness of drug use, attitudes toward behavior change, and ambivalent attitudes toward drug dependence. There are 19 items, and the items on the scale are rated on a 5-point Likert scale (each item ranging from “strongly disagree” to “strongly agree”). The questionnaire on motivation for abstaining from drugs is also used to evaluate treatment motivation. The scale has a total of 36 items, which are divided into the following 5 subdimensions: “tending to rehabilitation-internal motivation,” “tending to rehabilitation-external motivation,” “avoiding abuse-internal motivation,” “avoiding abuse-external motivation,” and “confidence in abstaining from drugs.” The total score of treatment motivation is summed up by the scores of each dimension. The higher the scores, the stronger the treatment motivation.

Visual analogue scale (VAS) is used to assess the self-perception of the importance of drug abstinence (simply called “IMPORTANCE”), their confidence in stopping drug use (simply called “CONFIDENCE”), and their psychological craving for drug use (simply called “CRAVING”). The VAS scale ranges from 0 mm to 100 mm.

For the items of “IMPORTANCE” and “CONFIDENCE,” “0” means “doesn’t matter at all,” and “100” means very important. For “CRAVING,” “0” corresponds to “no craving,” and “100” represents “highest craving intensity ever experienced for drug.”

The validity and reliability of the Chinese version of the following scale have been confirmed previously: GAD-7, PHQ-9, SOCRATES, BIS-11, and questionnaire of motivation for abstaining from drugs [24-28].

During the assessment, the virtual digital psychotherapist reads the questions, and the patients select options on the tablet screen.

### Treatment Module

The treatment module of Echo-APP can provide patients with a variety of treatment options, aiming to help patients enhance their motivation to stop drug use, reduce drug cravings, strengthen self-control to avoid relapse, strengthen emotional management skills, and enhance individual and social functions (Multimedia Appendix 2).

The whole module includes the following 10 treatment units: (1) strengthening the motivation of drug withdrawal, (2) recognition of drug cravings and incentives, (3) high-risk situations identification and coping skill, (4) dealing with negative cognition, (5) understanding of emotions, (6) stress management, (7) understanding of family conflicts, (8) preventing relapse, (9) mindfulness, and (10) awareness of positive attitude and well-being (Multimedia Appendix 3). Based on the patient's assessment results, the treatment unit that meets the needs of the patient’s condition will be selected to provide to the patient.

Each treatment unit follows a structured setting for CBT. At the beginning of each treatment unit, there will be a brief introduction to the treatment goals and themes of the unit. After that, it will enter the formal treatment session, in which various common tools of CBT [29,30] (eg, thinking record sheet, analysis sheet of drug use pros and cons, risk level evaluation sheet for external factor, alternative behavior selection sheet), mindfulness-based relapse prevention technique [31] (integrated into treatment unit 6, 8, and 9), motivational enhancement therapy [32] (integrated into treatment unit 1), and related knowledge popularization (eg, the damage of different drugs and the antecedent, behavior, and consequence theory of emotion) will be applied.

### Homework Module

The homework module corresponds to the treatment module. There are 10 themed homework modules. After each treatment unit is completed, patients will be assigned their homework and asked to complete it offline (Multimedia Appendix 4).

### Feedback

After completing each assessment, Echo-APP will provide patients with assessment reports and treatment recommendations. For each treatment unit, Echo-APP will first ask the patient about their treatment experience and their change (eg, treatment motivation and confidence to deal with negative emotions), and then provide the summary report of this treatment unit. The report form is shown in Multimedia Appendix 5.

### Instruction Booklet

Although the clinical assessment and treatment implemented in Echo-APP can be useful for patients with substance dependence, good guidance is still required for initial use. To this end, in order to make the use of Echo-APP more practical, safer, and more extensive, we provide the Echo-APP instruction manual (Chinese version) and will provide an English version in the future. With a user-friendly and tailored interface, Echo-APP is easy to learn, and this manual will be an effective tool for patient self-learning. This manual shows the interfaces you may encounter in Echo-APP and what you need to do to complete the interface for each assessment or treatment.

### Study Design

This study consists of 3 parts, the first of which describes the development and technical details of Echo-APP. Then, to assess the efficacy and feasibility of the Echo-APP–based assessment and treatment, we conducted a preliminary study in patients with methamphetamine dependence. Specifically, we conducted a single-arm self-control pilot study with the treatment unit “strengthening the motivation of drug withdrawal.”

### Ethical Considerations

This study was carried out in accordance with the principles of the Declaration of Helsinki and approved by the institutional review board and the ethics committee of the Shanghai Mental Health Center (approval number: 2020-92). The participants provided their informed consent before the study. The study flow diagram is shown in Multimedia Appendix 6.

### Participants

A total of 49 patients were recruited from the Shanghai Drug Rehabilitation Center. Eligible patients were diagnosed with methamphetamine use disorder by psychiatrists based on the Diagnostic and Statistical Manual of Mental Disorders, Fifth Edition (DSM-5) criteria. The inclusion criteria were as follows: (1) met the DSM-5 criteria for methamphetamine use disorder, (2) aged 18-55 years, and (3) normal or corrected-to-normal vision and audition. The exclusion criteria were as follows: (1) with severe cognitive deficits or impairments; (2) with serious physical or neurological illness or a diagnosis of any other psychiatric disorder under DSM-5 criteria (except for nicotine use disorder); and (3) inability to understand and operate the app instructions. Two patients stopped from participating in the study after the screening. Therefore, 47 patients were finally included in the analysis.

### Treatment Settings

All participants received one session of treatment unit, “strengthening the motivation of drug withdrawal” (Echo-APP–based MET), which was provided by a virtual digital therapist. The contents of the Echo-APP–based MET include an introduction to the damage of drugs, analysis and comparison of the pros and cons of drug abuse, sharing of stories and experiences of people who have successfully abstained from drugs, and improvement of motivation for change. The session duration is about 30 to 45 minutes. During the treatment, there will be a real psychotherapist familiar with the operation of Echo-APP to provide the necessary operation guidance for the patients. Other than that, the real psychotherapist does not provide any therapy during treatment.

### Outcome Measures

The primary treatment outcome was the change in SOCRATES score. The secondary outcomes include the following: (1) score change on the questionnaire of motivation for abstaining from drugs; (2) score change of the VAS items “IMPORTANCE,” “CONFIDENCE,” and “CRAVING.”

### Adverse Effect

During assessment and treatment, no participant reported significant discomfort or adverse effects.

### Statistical Analysis

To identify the treatment efficacy of Echo-APP, paired 2-tailed *t* test was used to analyze the changes in scale scores before and after treatment. For those clinical outcomes that have changed significantly before and after treatment (ie, SOCRATES, questionnaire of motivation for abstaining from drugs, IMPORTANCE, CONFIDENCE, and CRAVING), the general linear regression model was used to analyze potential factors affecting treatment efficacy. For each of the general linear regression models, the change in the scale scores (ie, SOCRATES, questionnaire of motivation for abstaining from drugs, IMPORTANCE, CONFIDENCE, and CRAVING) was used as the dependent variable, and baseline demographic information (ie, age, education, current marital status), drug use history, emotional state (ie, GAD-7 and PHQ-9), and impulsivity characteristics (ie, BIS-11) were included as independent variables in the model. The backward method was used to screen the independent variables. All the above statistical analyses were finished using SPSS 20.0 (IBM Corp).

## Results

### Baseline Information

Baseline demographic characteristics and clinical data are presented in Table 1. The average age of the patients is 38.85 (SD 8.08) years. The patients’ accumulated months of methamphetamine use is 99.89 (SD 56.71) months. Of the 47 participants, 12 (26%) use methamphetamine due to psychological craving.

**Table 1:** Demographic information and drug use history for all participants (N=47) in a study of Echo-APP–based Motivational Enhancement Therapy for methamphetamine use disorder.

Characteristics				Values
**Demographics**				
	Age (years), mean (SD)		38.85 (8.08)	
	**Education, n (%)**			
		Less than 7 years	1 (2)	
		7-9 years	18 (38)	
		10-12 years	17 (36)	
		More than 12 years	11 (23)	
	Currently married, n (%)		16 (34)	
**Drug use history**				
	Accumulated months of methamphetamine use, mean (SD)		99.89 (56.71)	
	Methamphetamine use dosage (grams) per day, mean (SD)		0.71 (0.42)	
	**Methamphetamine use frequency, n (%)**			
		Use every day	20 (43)	
		3-5 days per week	7 (15)	
		1 day per week	9 (19)	
		1-3 days per month	11 (23)	
	**Methamphetamine use reason, n (%)**			
		Craving	12 (26)	
		Others	35 (74)	
**Baseline clinical measures, mean (SD; range)**				
	Craving		18.09 (26.41; 0-100)	
	Awareness of the importance of drug abstinence		77.13 (32.47; 0-100)	
	Confidence in drug abstinence		83.09 (23.26; 0-100)	
	SOCRATES^a^		65.13 (15.23; 26-91)	
	Questionnaire of motivation for abstaining from drugs		160.79 (19.48; 109-180)	
	PHQ-9^b^		5.53 (4.84; 0-19)	
	GAD-7^c^		3.79 (4.26; 0-16)	
	BIS-MI^d^		73.13 (17.73; 34-100)	

^a^SOCRATES: Stages of Change Readiness and Treatment Eagerness Scale.

^b^PHQ-9: Patient Health Questionnare-9.

^c^GAD-7: Generalized Anxiety Disorder-7.

^d^BIS-MI: Barratt Impulsiveness Scale—motor impulsivity.

### The Efficacy of Echo-App–Based Motivational Enhancement Therapy

**Figure 2:**
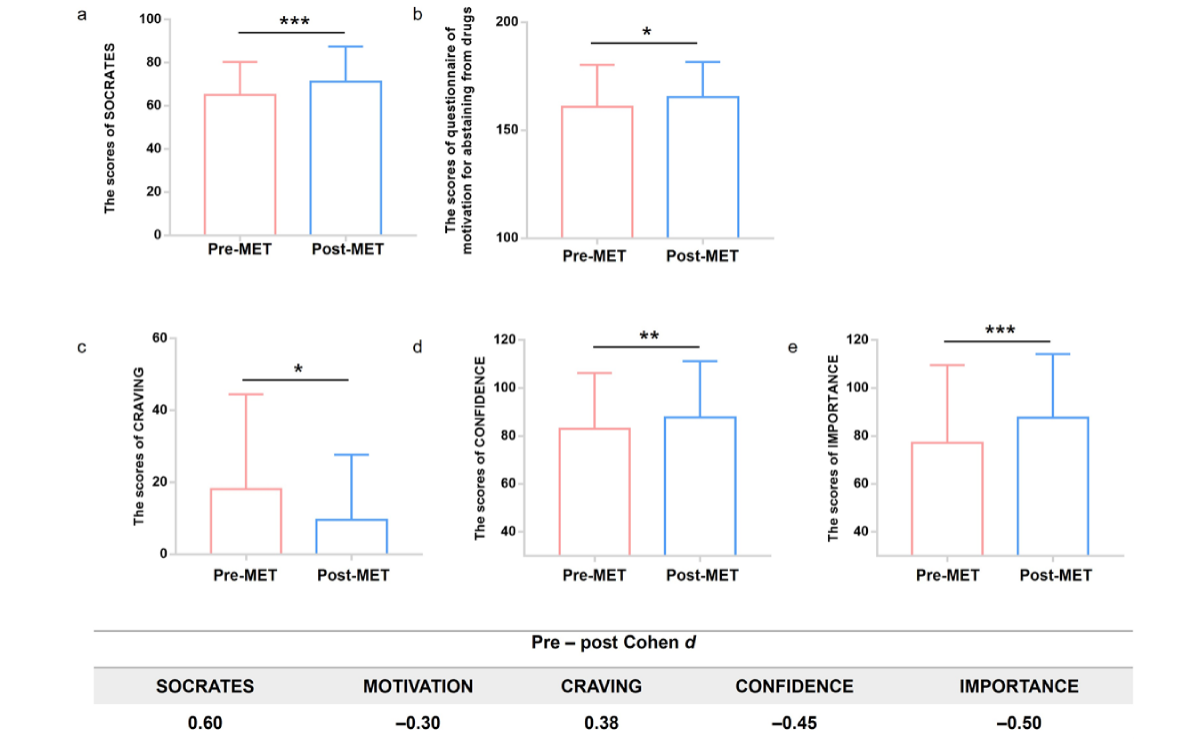
The changes of the clinical measures and effect sizes (Cohen d) during the Echo-APP–based motivation enhancement treatment (MET). (a) The changes of the Stages of Change Readiness and Treatment Eagerness Scale (SOCRATES) scores. (b) The changes of the questionnaire of motivation for abstaining from drugs. (c) The changes of visual analogue scale score of “CRAVING.” (d) The changes of visual analogue scale score of “CONFIDENCE.” (e) The changes of visual analogue scale score of “IMPORTANCE.” *<.05, **<.01, ***<.001.

The Echo-app–based MET treatment has brought significant improvement in patients’ treatment motivation as assessed on the SOCRATES scales (*P*<.001, Cohen *d*=–0.60) and the questionnaire of motivation for abstaining from drugs (*P*=.045, Cohen *d*=–0.30). Besides, it had a significant effect on the reduction of self-reported craving (*P*=.01, Cohen *d*=0.38; Figure 2 and Multimedia Appendix 7).

### Factors Affecting Echo-APP–Based Treatment Efficacy

**Figure 3:**
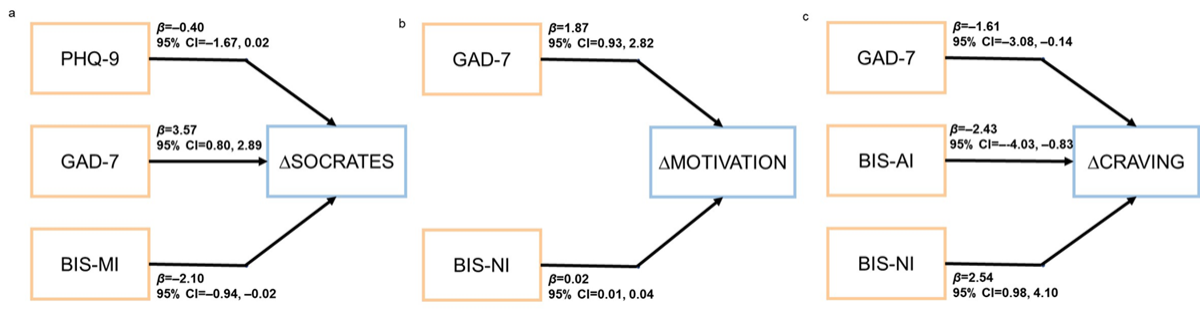
The regression analyses predicting change of clinical measures in patients with methamphetamine dependence. (a) Regression analyses predicting an increase in Stages of Change Readiness and Treatment Eagerness Scale (SOCRATES) score. (b) Regression analyses predicting an increase in score of questionnaire of motivation for abstaining from drugs. (c) Regression analyses predicting a decrease in craving score. BIS-AI: Barratt Impulsiveness Scale—attention impulsivity; BIS-MI: Barratt Impulsiveness Scale—motor impulsivity; BIS-NI: Barratt Impulsiveness Scale—nonplanning impulsivity; GAD-7: Generalized Anxiety Disorder; PHQ-9: Patient Health Questionnare-9.

To explore if the patients’ baseline characteristics may influence the treatment efficacy of Echo-APP–based MET, we investigated their associations. For the treatment motivation, the baseline GAD-7 score (*β*=3.57; *P*<.001; 95% CI 0.80, 2.89) and BIS—motor impulsivity scores (*β*=–2.10; *P*=.04; 95% CI –0.94, –0.02) were predictive of SOCRATES change after MET, and the baseline GAD-7 score (*β*=1.87; *P*<.001; 95% CI 0.93, 2.82) and abstinence duration (*β*=0.02; *P*=.01; 95% CI 0.01, 0.04) were predictive of questionnaire of motivation for abstaining from drugs change after MET (Figure 3).

For psychological craving, the baseline GAD-7 score (*β*=–1.607; *P*=.03; 95% CI –3.08, –0.14), BIS—attentional impulsivity scores (*β*=–2.43; *P*=.004; 95% CI –4.03, –0.83), and BIS—nonplanning impulsivity (*β*=2.54; *P*=.002; 95% CI 0.98, 4.10) were predictive of self-reported craving reductions after MET.

None of the baseline characteristics were relative to the changes in the visual analogue scale score of “CONFIDENCE” and “IMPORTANCE.”

## Discussion

### Overview

The purpose of this study was to describe the design of Echo-APP, which is the virtual digital psychotherapist app, and conduct a preliminary evaluation of the efficacy of the Echo-APP–based intervention. The study found that 1 session of Echo-APP treatment can enhance patients’ treatment motivation and reduce psychological cravings.

### The Design of Echo-APP

The core work of this study was to build standardized self-assessment and high-quality psychotherapy tools that are in line with the needs of patients with SUD. Therefore, medical staff and patients can use this professional digital tool for SUD treatment more conveniently and with more interest. Based on this consideration, we refer to the opinions of patients with SUD, discuss existing digital health programs with doctors, psychotherapists, and computer experts, and design new digital health program for patients with SUD. Our group mainly focuses on several points, including the following: (1) accessibility and convenience of the digital program, (2) standardization and effectiveness of assessment and treatment, and (3) optimizing the user-program interaction experience and enhancing immersion. Previous studies have found that both web-based and app-based text conversational agents are effective for patients [12], and app-based programs have advantages in improving accessibility [33]. However, dialogue agent tools that use text or animation to interact have their disadvantages, such as poor interaction and immersion. Besides, most digital programs also offer fewer treatment options, and patients are passively accepted [34]. In this context, we developed the first virtual digital psychotherapist program, Echo-APP, for patients with SUD, which is a more economical and interactive app that runs on the tablet platform.

This study describes the design and development process of Echo-APP and evaluates the utility of Echo-APP in a single-arm design study. From a technical point of view, Echo-APP provides a more friendly operation interface. Patients are able to complete the assessment and treatment process through interaction with a virtual digital psychotherapist. According to the needs of patients with SUD, Echo-APP has 10 units, which can provide patients with comprehensive treatment. Throughout the assessment and treatment process, Echo-APP provides readable feedback reports for patients, doctors, and psychotherapists to clearly know the patient's condition changes and treatment efficacy.

### Treatment Efficacy of Echo-APP

This study suggested that a single session of Echo-APP treatment can enhance patients’ treatment motivation and reduce psychological cravings. The design of this Echo-APP–based MET program is referred to the MET protocol of our group, which was found to significantly improve the treatment outcome of patients who are dependent on heroin [35]. Echo-APP also had an effect on reducing psychological cravings, which may be related to the improvement of treatment motivation [36]. To explore the factors that may affect the treatment effect, we analyzed the patient's baseline characteristics, and the results suggested that the patient's emotional state and impulsive personality characteristics may affect the effectiveness of the Echo-APP-based MET treatment. Therefore, the multi-unit comprehensive intervention system provided by Echo-APP may have better applicability. It is possible to improve the overall treatment effect through targeted intervention for different symptoms, which has been verified in other studies [37,38]. Although the results are promising, this work is a preliminary study, and further standardized evaluation of Echo-APP is still needed, and a multicenter randomized controlled clinical study is required to explore the efficacy and adverse reactions of Echo-APP.

### Limitations

This study inevitably has some limitations. Firstly, the current assessment of Echo-APP is mainly based on scales, which may have similar shortcomings as paper scales. We plan to develop an effective addiction symptom assessment paradigm to reduce the subjective assessment content. Secondly, this study is a single-arm design, which can only provide a preliminary result of the therapeutic value of Echo-APP, whereas the most rigorous way to ensure the effectiveness of the developed APP would be to conduct a randomized controlled clinical study in comparison with a traditional psychotherapy. This randomized study is in progress and in its early stages. Thirdly, this study is only conducted on patients with methamphetamine use disorder, and the popularity of the study results needs to be improved, including further research on people who use legal drugs such as tobacco and alcohol.

### Conclusion

In this study, we introduced the design and development of Echo-APP and preliminarily validated the efficacy of the Echo-APP–based treatment for patients with methamphetamine use disorder. This work fills the current lack of addiction treatment programs with the virtual digital psychotherapist on the market. In the future, we will continue to optimize the function of Echo-APP and carry out large-sample multicenter clinical controlled studies to verify the effect of Echo-APP and benefit more patients with SUD.

## Appendix

Multimedia Appendix 1 The interface of the assessment module.

Multimedia Appendix 2 The interface of the treatment module.

Multimedia Appendix 3 Ten treatment units in the treatment module.

Multimedia Appendix 4 The interface of the homework module.

Multimedia Appendix 5 The feedback report form provided by Echo-APP.

Multimedia Appendix 6 Flow diagram of the study.

Multimedia Appendix 7 The changes of the clinical measures during the Echo-app–based motivation enhancement treatment.

## Abbreviations

AI: artificial intelligence
BIS: Barratt Impulsiveness Scale
CBT: cognitive behavioral therapy
DSM-5: Diagnostic and Statistical Manual of Mental Disorders, Fifth Edition
GAD-7: Generalized Anxiety Disorder-7
MET: motivational enhancement therapy
PHQ-9: Patient Health Questionnaire-9
SOCRATES: Stages of Change Readiness and Treatment Eagerness Scale
SUD: substance use disorder
VAS: visual analogue scale
