# Supplementary figures and images for: Virtual Digital Psychotherapist App–Based Treatment in Patients With Methamphetamine Use Disorder (Echo-APP): Single-Arm Pilot Feasibility and Efficacy Study
Source: JMIR Mhealth Uhealth. 2023 Jan 31;11:e40373. doi: 10.2196/40373 (PMC9929731; doi:10.2196/40373)

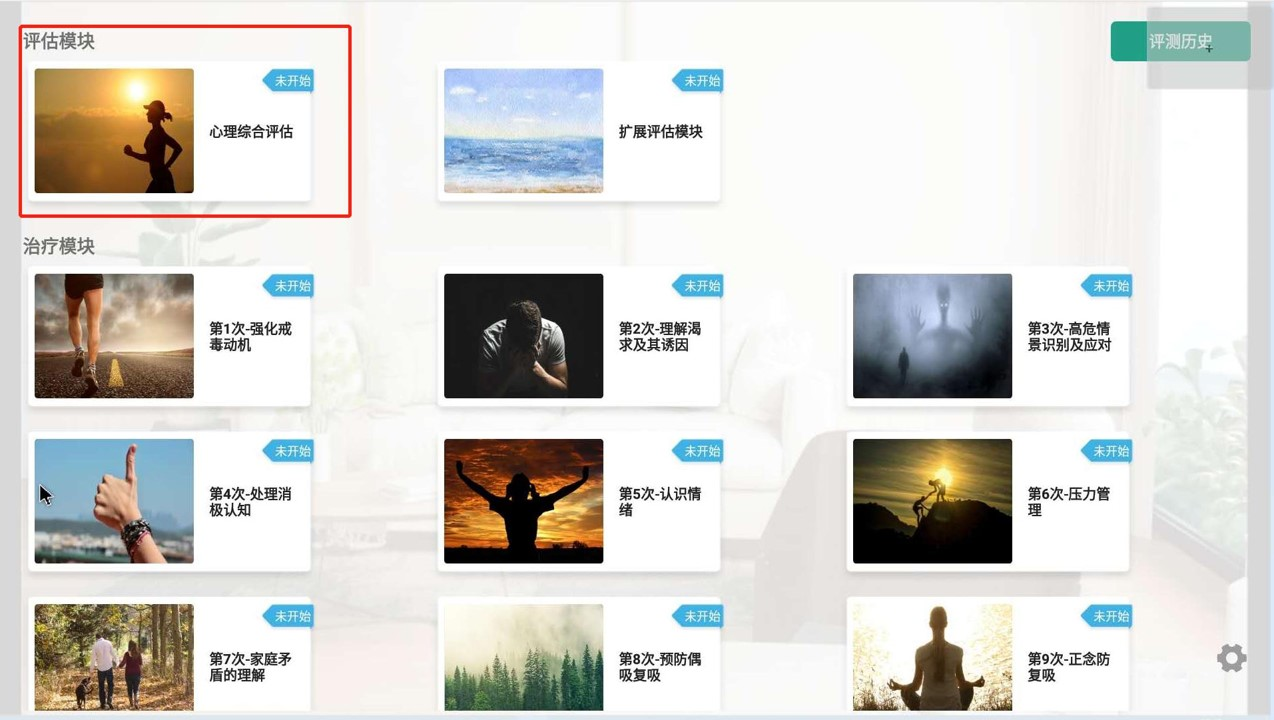

Supplement: Multimedia Appendix 1 [file mhealth_v11i1e40373_app1.png]

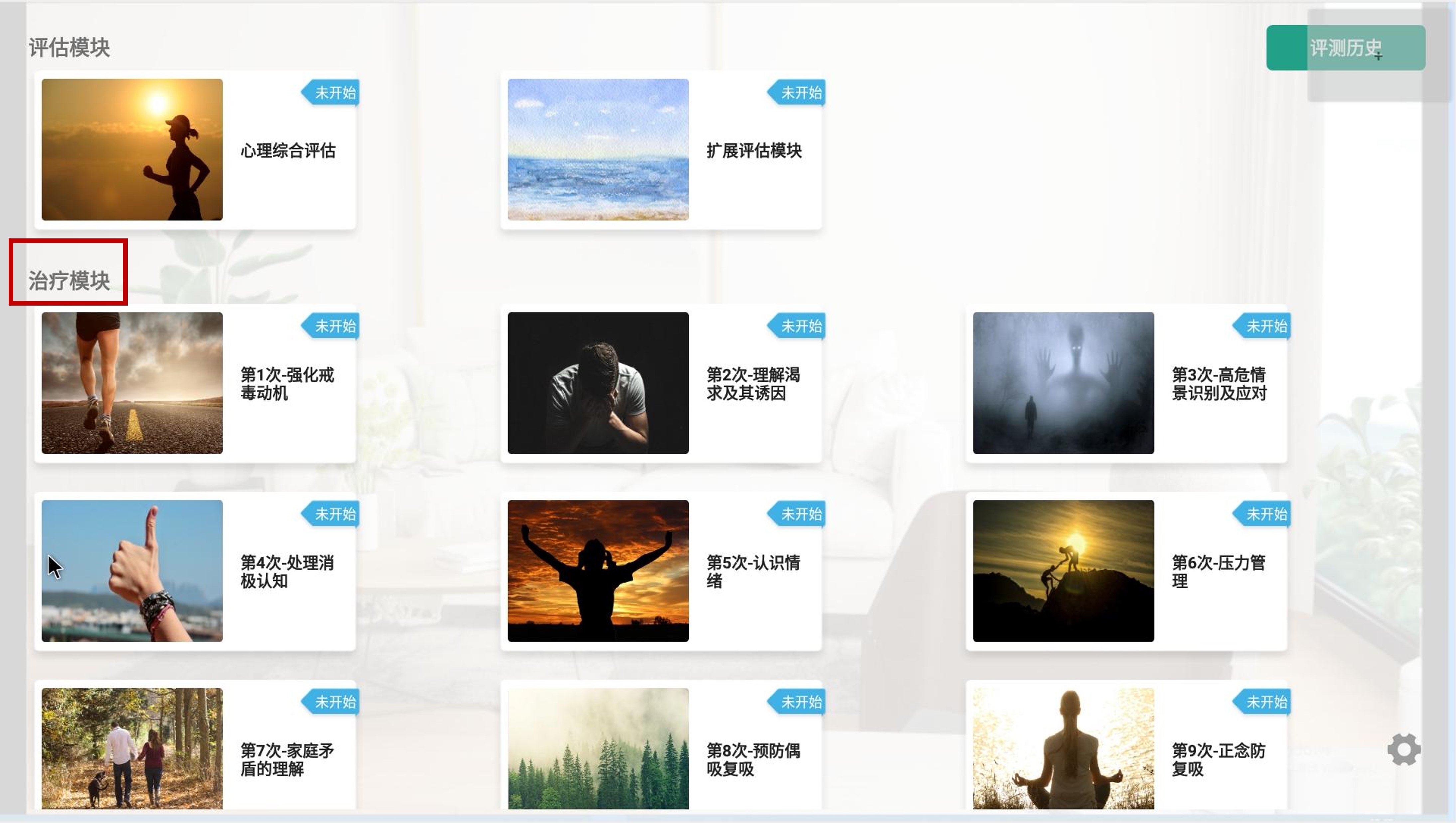

Supplement: Multimedia Appendix 2 [file mhealth_v11i1e40373_app2.png]

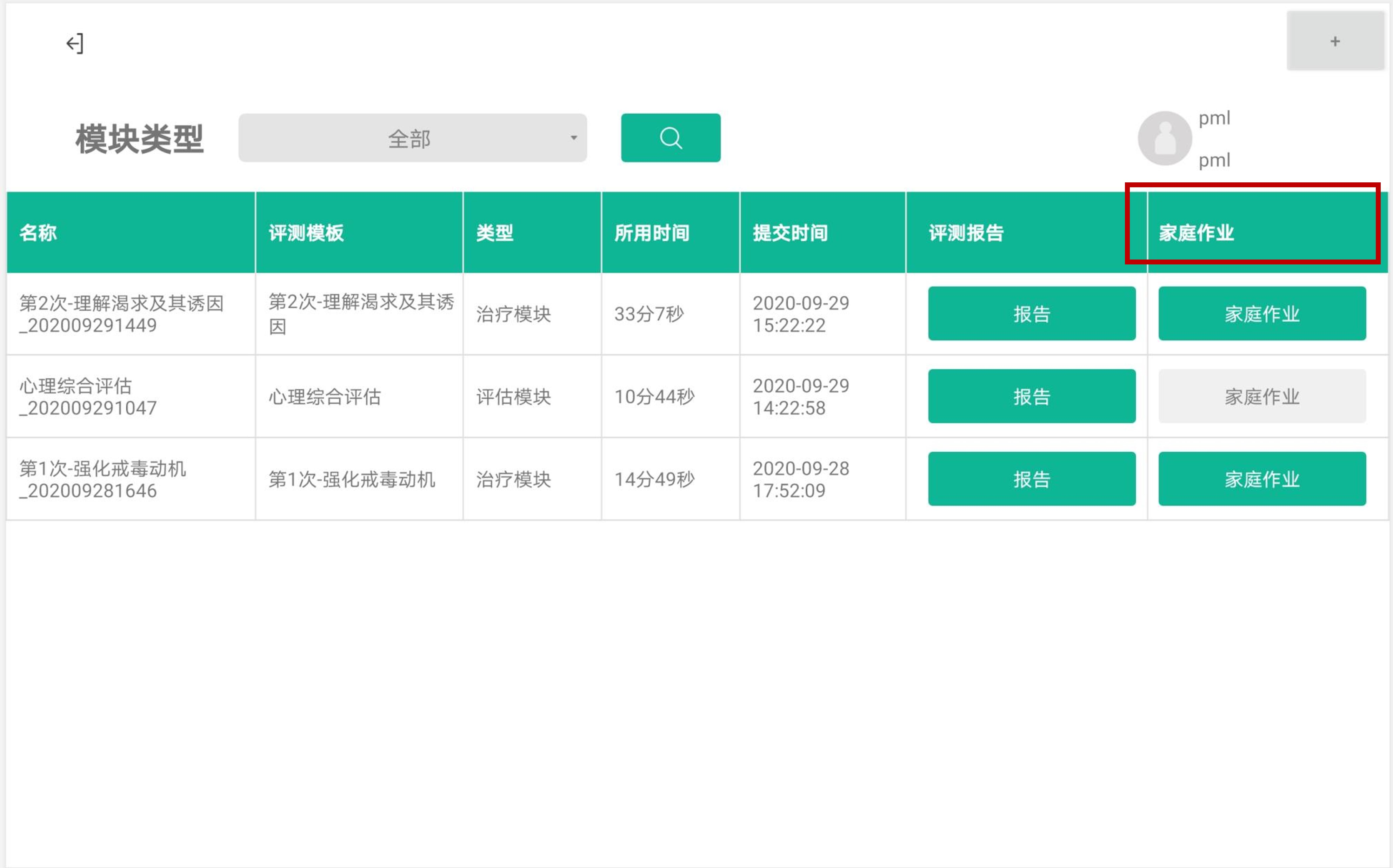

Supplement: Multimedia Appendix 4 [file mhealth_v11i1e40373_app4.png]

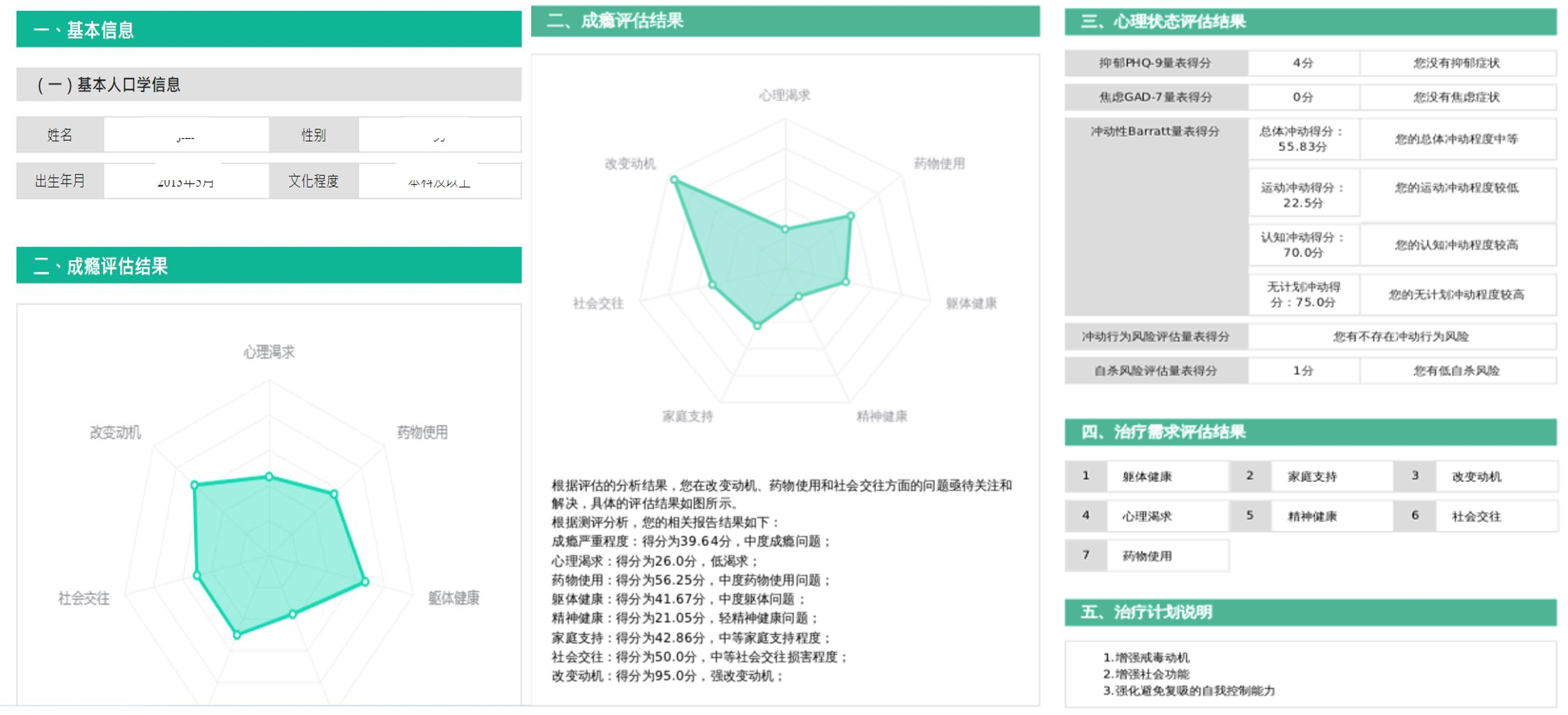

Supplement: Multimedia Appendix 5 [file mhealth_v11i1e40373_app5.png]

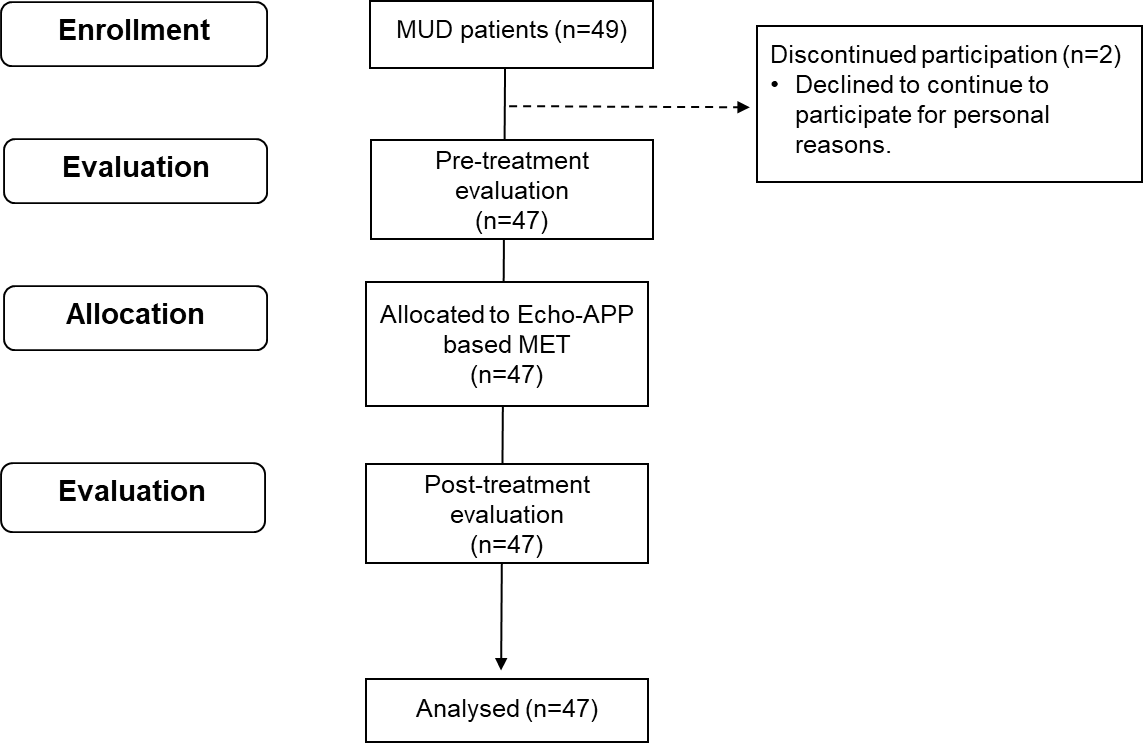

Supplement: Multimedia Appendix 6 [file mhealth_v11i1e40373_app6.png]
