# Supplementary material for: Virtual Digital Psychotherapist App–Based Treatment in Patients With Methamphetamine Use Disorder (Echo-APP): Single-Arm Pilot Feasibility and Efficacy Study
Source: JMIR Mhealth Uhealth. 2023 Jan 31;11:e40373. doi: 10.2196/40373 (PMC9929731; doi:10.2196/40373)
Supplement: Multimedia Appendix 3 [file mhealth_v11i1e40373_app3.docx]

|  | | |
| --- | --- | --- |
| Treatment Unit | Theme | Description |
| Unit 1 | Strengthening the motivation of drug withdrawal | This treatment unit is designed to help patients increase their motivation to stop drug abuse. The harm and differences of various drugs will be introduced. This treatment unit will also introduce the stories of inspirational characters and the personal experiences of people who successfully stop drug abuse, therefore, to resonate with patients and encourage patients to challenge themselves. In addition, useful tools such as “analysis sheet of drug use pros and cons” are recommended to help patients take their first step towards change. |
| Unit 2 | Recognition of drug cravings and incentives | This treatment unit is designed to help patients reduce their drug cravings.  In this unit, the virtual digital psychotherapist will introduce various potential incentives, and ask patients about their previous drug use-inducing experiences, help patients recall drug use situations, and complete their personal drug use situation record sheet. The neural mechanisms underlying cravings are explained and effective craving coping skills are provided, such as distraction, relaxation training, talking about cravings with others. |
| Unit 3 | High-risk situations identification and coping skill | This treatment unit is designed to help patients improve self-control ability to avoid relapse. The virtual digital psychotherapist will ask patients to identify the common high-risk scenarios for drug relapse and abuse, as well as instructs patients to fill in the external triggering factors and risk rating sheet. This unit will provide patients with psychological skills for coping with high-risk situations, like the SOBER breathing space exercise, alternative behavior methods. |
| Unit 4 | Dealing with negative cognition | Designed to help patients improve their emotional management skills. This treatment unit will explain the ABC theory of emotions to the patient, introduce the association between events, thoughts, and emotions. Besides, the common types of cognitive errors that affect thinking will also be introduced. Virtual psychotherapist will help the patient to fill out a record sheet about though change. |
| Unit 5 | Understanding of emotions | Designed to help patients improve their emotional management skills. This treatment unit will introduce the concept of emotion management, and guide patients to be aware of their emotions at the present moment, and then propose methods and tools for managing emotions based on the awareness of emotions. |
| Unit 6 | Stress management | Designed to help patients enhance their personal and social functioning. This treatment unit will introduce the knowledge of stress, including the source of stress and the characterization of stress in different stages. Patients are then told the potential association between drug use and stress, and how to perceive stressful situations. Finally, help patients cope with stress through tools such as stress event sequencing, resilience improvement, and meditation relaxation. |
| Unit 7 | Understanding of family conflicts | Designed to help patients improve their personal and social functioning. This treatment unit focuses on family conflicts, proposes a new perspective on family issues, and helps patients redefine family relationships and their possible conflicts in the past. Defense mechanisms are also introduced to further explain the relationship between family members. |
| Unit 8 | Preventing relapse | Designed to help patients strengthen their self-control to avoid relapse. This unit will first introduce the internal and external causes of relapse and allow patients to complete their own analysis of potential reasons for relapse. Then the virtual digital psychotherapist will introduce the key factors affecting relapse. Finally, help the patient to learn the skills of coping with relapse, such as making early warning cards, weighing the advantages and disadvantages of drug use. |
| Unit 9 | Mindfulness | Designed to help patients improve their emotional management skills. This treatment unit will systematically introduce the background and history of mindfulness, and through meditation exercises such as "Mountain Meditation" and "Sit-In Meditation", patients will have a deeper understanding of the use and feeling of mindfulness. |
| Unit 10 | Awareness of positive attitude and well-being | Designed to help patients improve their personal and social functioning. This treatment unit will promote patient self-awareness and introduce discussion about well-being. For instance, recognizing different types of happiness, learn skills and methods to improve happiness, and the concept of mandala flowers. |
