# Supplementary material for: Virtual Digital Psychotherapist App–Based Treatment in Patients With Methamphetamine Use Disorder (Echo-APP): Single-Arm Pilot Feasibility and Efficacy Study
Source: JMIR Mhealth Uhealth. 2023 Jan 31;11:e40373. doi: 10.2196/40373 (PMC9929731; doi:10.2196/40373)
Supplement: Multimedia Appendix 7 [file mhealth_v11i1e40373_app7.docx]

| **Clinical Measures** | **Pre-intervention** | **Post-intervention** | **t value** | **P value** | **Cohen’s d** |
| --- | --- | --- | --- | --- | --- |
| Craving (mean, SD) | 18.09 (26.41) | 9.62 (18.12) | 2.59 | .013 | 0.38 |
| Awareness of the importance of drug abstinence (mean, SD) | 77.13 (32.47) | 87.62 (26.54) | -3.46 | <.001 | -0.50 |
| Confidence in drug abstinence (mean, SD) | 83.09 (23.26) | 87.79 (23.38) | -3.06 | .004 | -0.45 |
| SOCRATES (mean, SD) | 65.13 (15.23) | 71.19 (16.38) | -4.12 | <.001 | -0.60 |
| Questionnaire of motivation for abstaining from drugs (mean, SD) | 160.79 (19.48) | 165.32 (16.31) | -2.06 | .045 | -0.30 |
| SOCRATES= Stages of change readiness and treatment eagerness scale. | | | | | |
